# Food Supplements in Osteoarthritis: A Practical Framework for Discussing Evidence with Patients

**DOI:** 10.3390/nu18152561

**Published:** 2026-08-05

**Authors:** Matteo Briguglio, Thomas W. Wainwright

**Affiliations:** 1 IRCCS Ospedale Galeazzi-Sant’Ambrogio, Laboratory of Nutrition Sciences, 20157 Milan, Italy; 2 Orthopaedic Research Institute, Bournemouth University, Bournemouth BH12 5BB, UK; 3 University Hospitals Dorset NHS Foundation Trust, Bournemouth BH7 7DW, UK; 4 Lanzhou University, Lanzhou 730000, China

**Keywords:** dietary supplementations, nutraceuticals, osteoarthritis, complementary therapies, quality of health care, orthopaedic surgery, patient reported outcome measures, nutrition therapy, enhanced recovery after surgery, professional patient relationship

## Abstract

The treatment of osteoarthritis (OA), a leading cause of disability worldwide, is increasingly integrating nutritional strategies that appear to offer the opportunity to significantly improve the quality of care. Patients are also aware of this and often seek clarification and advice from OA professionals. However, it is still too early to formulate definitive recommendations, as the scientific evidence is still heterogeneous. This can create a gap between patients’ need for clear guidance and the necessarily prudent communication of professionals. As a result, some patients may make decisions on their own, forgoing the opportunity to receive nutritional care personalised according to their dietary habits and disease severity. This commentary presents an expert interpretation of the currently available evidence and proposes a practical framework to support clinical discussions with patients. It is not intended as a formal clinical practice guideline but aims to help OA professionals navigate conversations about the role that nutritional strategies may play in disease management.

## 1. The Practical Needs of Healthcare Professionals

Patients with osteoarthritis (OA) frequently ask healthcare professionals whether food supplements such as collagen, turmeric or glucosamine are worthwhile. Although interest in these products continues to grow, clinicians outside nutrition specialties often lack practical resources to guide these conversations.

Osteoarthritis is a disease that can affect any joint and is characterised by metabolic and structural changes that affect the entire joint unit, involving cartilage, bone, ligaments, and surrounding tissues. Historically, OA was defined as a wear-and-tear disease, as the aetiological factors responsible for its onset and progression were believed to be predominantly mechanical in nature. These include musculoskeletal abnormalities, characterised by joint misalignment, or excess body weight, which over time leads to local overload stress [1]. Indeed, the most frequently affected sites are the semi-mobile joints of the spine and the mobile joints of the hip and knee. Today, however, we know that the aetiology of OA is multifactorial, including chronic low-grade inflammation, muscle weakness, environmental factors, and genetic predisposition [2]. OA has a significant impact on patients’ daily lives because, depending on the severity of the disease, it can manifest itself with joint pain, stiffness of the limb or of the affected anatomical region, loss of mobility, and progressive limitation of daily activities.

Since there is no definitive cure, treatment is based on a combination of non-pharmacological approaches, anti-inflammatory drugs, intra-articular injections, additional pharmacological treatments, and surgery. Non-pharmacological approaches include physiotherapy and patient education on healthy lifestyle habits. Among the interventions involving nutrition, the best known is adherence to a healthy and balanced diet aimed at weight management. At the same time, the use of food compounds in the form of supplements is becoming increasingly widespread. These, thanks to their natural presence in the joints or their ability to modulate inflammatory processes, could help reduce pain and slow the progression of the disease.

However, while OA patients frequently receive physiotherapy from dedicated professionals, nutritional counselling is offered less frequently. The limited availability of this service is compounded by the caution with which national bodies, scientific societies, and patient associations express themselves regarding the use of food supplements. The Osteoarthritis Research Society International recommends dietary weight management when indicated but recommends against the use of chondroitin sulphate, vitamin D, and collagen [3]. Similarly, the American College of Rheumatology and the Arthritis Foundation recommend against glucosamine and fish oil [4]. In contrast, the European Alliance of Associations for Rheumatology does not provide specific recommendations regarding food supplements for OA [5], whereas the European Society for Clinical and Economic Aspects of Osteoporosis, Osteoarthritis and Musculoskeletal Diseases supports only the use of selected glucosamine and chondroitin formulations under specific circumstances [6]. These different positions can pose challenges for OA professionals, as the use of these food products among patients is widespread. For example, more than half report using glucosamine, and up to seven in ten have tried fish oil [7]. Consequently, while ensuring safety and appropriateness, clinicians should also manage patients’ expectations. It is not uncommon for professionals to be asked for opinions on the efficacy of glucosamine or the benefits from collagen and turmeric. It is therefore essential to be able to provide balanced, evidence-based answers, without raising false expectations or compromising one’s professional credibility.

## 2. Conceptual Reorganisation of Nutritional Interventions

Based on the mechanism of action through which they contribute to clinical benefit in OA, five categories of nutritional interventions can be identified:

1. interventions that aim to modulate inflammation, for example by reducing the production of pro-inflammatory cytokines, resulting in pain reduction;
2. interventions that help preserve joint structures by maintaining the integrity of cartilage, the extracellular matrix, and joint elasticity;
3. interventions targeting the bone-cartilage interface, whose endocrine effects aim to positively influence subchondral bone remodelling;
4. interventions supporting the muscle–cartilage interface, whose endocrine effects can improve muscle strength and mobility of the affected limb;
5. interventions aimed at cellular efficiency, which affect cellular metabolism and oxidative stress.

Most nutritional strategies proposed for OA, regardless of the level of evidence supporting their efficacy, fall into the first two categories. This reflects the fact that many of the molecular mediators involved in OA pathogenesis exert both pro-inflammatory and catabolic effects on joint tissues.

Examples of food compounds that fall into the first category are the omega-3 polyunsaturated fatty acids, derived primarily from fish or krill oil, and various bioactive compounds of plant origin. These include derivatives of plants from the Zingiberaceae family, such as gingerols from ginger and curcumin from turmeric, polyphenols extracted from berries, boswellic acid derived from species of the *Boswellia* genus, as well as compounds found in garlic and chilli peppers. The second category includes both dietary interventions aimed at reducing body weight and thus joint overload, as well as supplementation strategies that involve the use of collagen, glucosamine, chondroitin sulphate, methylsulfonylmethane (MSM), vitamin K [8] and S-adenosyl-L-methionine (SAM) [9]. Collagen can be extracted from chicken sternum, while chondroitin sulphate can be obtained from bovine trachea. The third category includes calcium and vitamin D, the fourth category includes proteins, amino acids, and magnesium, while the fifth category includes L-carnitine and various plant-based bioactive compounds, including those derived from cinnamon.

The five-category framework proposed here is an original, expert-derived conceptual model intended to facilitate clinical discussion of nutritional interventions in OA. Although useful from a practical perspective, it simplifies the complex and often overlapping biological mechanisms through which nutritional interventions may exert their effects and has not been formally validated. It is therefore important to emphasise that a single intervention can act through multiple processes simultaneously. For example, reducing body fat through diet produces benefits both by reducing mechanical load on joints (category 2) and by reducing chronic low-grade inflammation (category 1). Similarly, vitamin D influences both calcium homeostasis (category 3) and muscle metabolism (category 4).

Further examples of compounds characterised by multiple mechanisms of action are capsaicin, hyaluronic acid [10] and probiotics [11]. Capsaicin, the main bioactive compound of chilli pepper, is used as a food supplement for its anti-inflammatory properties (category 1), but also and above all as a topical preparation with analgesic action. Hyaluronic acid, naturally present in the body and contained in some foods, is a component of the extracellular matrix of cartilage and is used both as a food supplement and as an intra-articular treatment for the benefit of joint structures (category 2). Finally, some probiotic strains could exert beneficial effects both through the modulation of inflammation (category 1) and through the production of vitamin K (category 2).

## 3. Nutritional Priorities

Science is based on the continuous testing of hypotheses that progressively approach trueness yet never reach it definitively. Communicating this process to patients is complex, and inevitably requires addressing concepts such as expectations, efficacy, safety, costs, and individual response variability. Patients, however, tend to seek simple, binary answers. Those who ask whether a particular food supplement is beneficial often expect a straightforward, affirmative or negative answer.

A disapproving response without adequate explanation could compromise the professional’s credibility. Conversely, uncritical approval could fuel unrealistic expectations regarding an intervention that could prove ineffective or, in some cases, risky. A fundamental principle, therefore, remains that of doing no harm, and food supplements should not be assumed to be free of safety risks. For example, glucosamine has been associated with concerns regarding glucose metabolism in some individuals [4] and poorer outcomes among patients with dementia [12], although robust evidence of causality is still lacking. In most cases, the most appropriate strategy is likely to reorient the patient’s priorities.

When asked for an opinion on a specific nutritional strategy for OA, it is important to understand the rationale. This allows us to assess the patient’s level of knowledge, expectations, past experiences, information sources used, and any dissatisfaction with other non-pharmacological interventions already in place. Understanding whether the patient is primarily seeking pain relief, disease progression modification, or overall health improvement is an essential starting point for the discussion. Second, it is helpful to clarify that nutritional strategies can be conceptualised as a hierarchy for OA. At the base is a healthy and balanced diet (first-level interventions), at the middle level is body fat management (second-level interventions), and at the top are nutritional supplements (third-level interventions). Supplements, as the term itself suggests, are intended to complement, not replace, first-level and second-level interventions.

It follows that a healthy, balanced diet naturally promotes achieving and maintaining an appropriate body weight. Communicating that healthy eating, in synergy with physical exercise, represents one of the pillars of non-pharmacological management of OA is therefore of fundamental importance. This concept was recently reaffirmed by an umbrella review [13], helping to strengthen the role of diet, and in particular the Mediterranean diet [14], in the management of OA compared to strategies based exclusively on food supplements.

In patients who are sedentary, physically inactive, with unhealthy dietary habits, overweight or obese and with poor pain control, the request for a food supplement ought to represent an opportunity to reconsider nutritional priorities, thus bringing attention back to fundamental interventions.

## 4. Interpreting the Scientific Evidence

Once priority nutritional interventions have been consolidated, it may be appropriate to address the complementarity of food supplements. Generally, these concentrated sources are taken orally in the form of tablets, capsules, powders, or liquid preparations. They may contain nutrients, such as vitamins or essential fatty acids, non-nutrients, such as bioactive plant compounds, or a combination of both.

The legislation governing the labelling of food supplements and foods for special medical purposes only permits the use of health claims supported by adequate scientific evidence. In Europe, such claims are governed by Regulation (EU) No 1924/2006 of the European Parliament and of the Council. Since the marketing of products making health claims not supported by scientific evidence is prohibited, encouraging patients to carefully read labels can be a first step towards patient activation and protection. It is also possible to consult the public register of health claims authorised by the European Commission (ec.europa.eu/food/food-feed-portal/screen/health-claims/eu-register), previously evaluated by the European Food Safety Authority (EFSA). To date, the main nutrients potentially relevant to the management of OA that have received a favourable assessment by EFSA include proteins, vitamin C, vitamin D, vitamin K, calcium, copper, manganese, magnesium, phosphorus, and zinc. The authorised claims primarily concern the maintenance of healthy bones, cartilage, and connective tissue. It is therefore not surprising that these nutrients are frequently included in commercial formulations intended for OA patients. It is important to note that the recognised effects depend not only on the presence of the nutrient, but also on achieving specific minimum amounts in the recommended daily intake. Conversely, several health claims potentially relevant to OA have not been approved. These include those related to avocado and soy extracts, chondroitin, collagen, curcumin, glucosamine, hyaluronic acid, krill oil, MSM, omega-3 fatty acids, SAM, and shark cartilage. This lack of approval does not necessarily imply a lack of benefit but rather reflects insufficiency of the evidence available at the time of the assessment. Regulatory requirements and authorised health claims for food supplements vary across jurisdictions. Therefore, the EFSA framework described here may not be directly applicable outside Europe. Furthermore, as this is a commentary rather than a systematic review, the literature was not identified through a predefined systematic search. We purposively selected recent systematic reviews and meta-analyses of randomised controlled trials covering commonly used food supplements and considered them alongside major international OA guidelines. The evidence presented should therefore be interpreted as a narrative, expert-informed overview rather than a comprehensive evidence synthesis. In addition to the EFSA evaluation of safety and efficacy, several published meta-analyses of randomised controlled trials have synthesised the available evidence on food supplements for OA, predominantly in patients with knee OA [11,15,16,17,18,19,20,21,22,23,24,25,26,27,28]. Promising, although not yet definitive, effects have been reported for vitamin D, omega-3 fatty acids, krill oil, *Lactobacillus* strains, eggshell membrane, collagen, chondroitin sulphate, glucosamine, *Boswellia*, passion fruit peel extract, ginger, and turmeric.

In light of this evidence and the considerations developed in the previous sections of this article, to translate scientific knowledge into clinical practice and feel confident in recommending a food supplement, practitioners should consider at least five aspects: patient adherence to first- and second-level strategies, the patient’s clinical condition, the stage of the disease, the product dosage, and the reliability of the available evidence.

Patient’s health is the primary factor to consider when evaluating the appropriateness of using a food supplement. Rather than guiding the choice towards a specific compound, the clinical condition can influence the risk–benefit profile of the intervention. For example, older individuals frequently experience conditions that can alter the response to supplements, including reduced nutrient intake, intestinal malabsorption, multiple comorbid conditions, and polypharmacy. The latter deserves particular attention because some supplements can interact with medications commonly prescribed in the older population, impacting their efficacy or increasing the risk of adverse events. Furthermore, certain nutritional strategies may be more relevant in the presence of conditions that often coexist with OA, such as sarcopenia, frailty, osteopenia, or osteoporosis. Therefore, the decision to recommend a supplement should be contextualised within a comprehensive nutritional assessment of the patient. For example, in patients with concomitant reduced muscle mass and function, nutritional interventions targeting the muscle–cartilage interface (category 4) could be of greater practical relevance, although this hypothesis requires further experimental confirmation.

Regarding the stage of OA, there are currently no consolidated guidelines that allow for personalising the choice of supplement based on the severity of the disease. However, it is plausible to hypothesise that the selection of the intervention, similarly to what occurs with some intra-articular therapies, may depend on the severity of the OA, the presence of joint effusion, and the patient’s activity level. For example, supplements primarily aimed at structural preservation (category 2) may be less useful in moderate or severe forms, while strategies focused on modulating inflammation or improving muscle function (categories 1 and 4) may be more relevant in these cases. The stage of OA is likely to influence the time it takes to observe a benefit and the period of supplementation, which is an aspect often asked by patients. Time to effect strongly depends on the outcome measure being considered, the study design, and the specific intervention evaluated. Regardless of the type of food supplement, some data suggest that pain is the first symptom to improve [28], with clinically relevant effects generally not observed before three months of treatment [24]. Other studies have reported a maximum improvement in stiffness within the first ten months, while pain reduction may peak between the tenth and twentieth months. Overall disease severity may also continue to improve beyond an average of twenty months of treatment [29].

Regarding dosage, this depends largely on the formulation used, which can significantly influence the bioavailability of the compound and, consequently, the required dose. In general, it is advisable to follow the label’s instructions regarding the recommended daily dose. Also, regarding nutritional compounds like calcium and vitamin D, it is always important to emphasise that their use is primarily indicated in the presence of insufficient dietary intake, increased nutritional needs, or ascertained deficiency [30]. Therefore, before recommending a nutritional supplement in individuals where a deficiency is suspected, it would be advisable to perform a blood test because the extent of the deficiency can dictate whether high-dose or low-dose supplementation is necessary [31]. This nutrient status check does not apply to non-nutritional compounds, for which their normal concentration in the body is unknown. This is a further factor of uncertainty when talking about the dose-benefit balance of herbal remedies or nutraceuticals. It cannot be ruled out that the natural evolution of the disease influences the perception of the supplement’s efficacy. This concept should be openly communicated to the patient. For this reason, it is essential to plan adequate clinical monitoring and systematically collect patient-relevant outcome measures.

In Table 1, the current evidence and practical considerations for the food supplements most commonly encountered in clinical practice are summarised. The column “Authors’ narrative interpretation of the evidence” reflects the authors’ qualitative expert appraisal, intended to support clinical discussions in the context of this commentary. It does not represent a formal certainty-of-evidence assessment and was not derived using a recognised evidence appraisal framework.

## 5. Communicating in Clinical Practice

**Figure 1:**
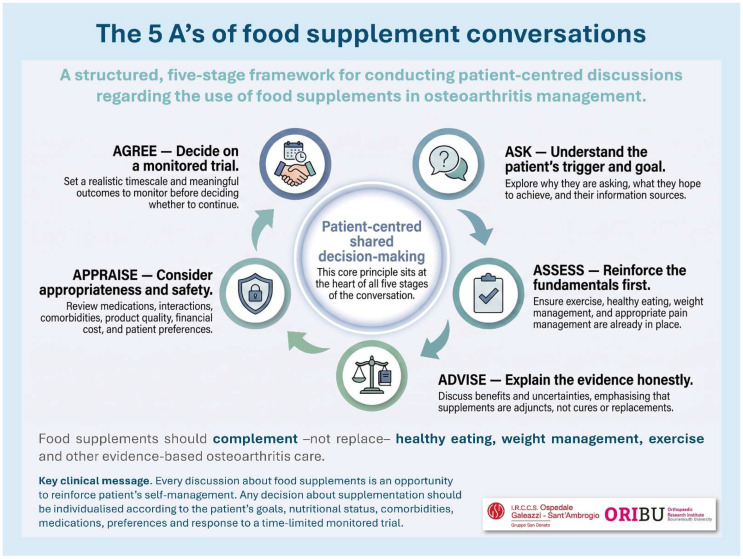
Flow-chart of the structured approach for patient visits when discussing food supplements.

To help OA professionals navigate conversations with patients about the role that certain nutritional strategies can play in disease management, we propose the 5 A’s of food supplements conversations (Figure 1 and Appendix A), a pragmatic framework designed to support clinicians when discussing the use of these products with patients. The framework is an expert-derived proposal developed by the authors for this commentary. Although the “5 A’s” format has precedent in behaviour-change and clinical counselling, the specific stages and content presented here were developed for conversations about food supplements in OA and have not yet undergone stakeholder consultation or evaluation in clinical practice.

In the current era of information overload, where medical information is readily available regardless of its veracity, patient requests for advice represent a unique opportunity to enhance the credibility of healthcare professionals regarding self-medication and protect patients from health risks. It is therefore important to be prepared to respond to topics outside of one’s academic background and area of expertise. Available evidence suggests that some nutritional strategies may contribute to managing OA symptoms more than others. It should be noted that the available clinical evidence is derived predominantly from studies in patients with knee OA, and the extent to which these findings apply to OA affecting other joints remains uncertain. Furthermore, there is heterogeneity of studies, high variability of formulations and doses, and lack of definitive evidence, which calls for caution when communicating with patients. In this context, the professional’s role is to verify that priority interventions, consisting of a healthy and balanced diet, weight management when necessary, and physical activity, have been addressed. Only then may it be appropriate to discuss the role of specific food supplements. As experienced OA professionals, with the 5 A’s of supplement conversations, we intend to provide a structured approach for patient visits. Future research should evaluate the feasibility, acceptability, and clinical impact of the proposed 5 A’s framework, including its effects on shared decision-making, patient satisfaction, and adherence. For non-nutritive bioactive compounds, the lack of a defined individual requirement makes assessing the safety profile particularly important, especially in the case of high dosages, contaminants, comorbidities, or concomitant therapies.

Several principles are therefore well established: food supplements should not replace exercise, healthy eating, weight management where indicated, or other evidence-based OA treatments. In contrast, the clinical effectiveness, optimal formulation, dose and duration of most individual supplements remain uncertain, with the available evidence derived predominantly from knee OA studies. Decisions about supplementation should therefore be individualised according to the patient’s goals, nutritional status, comorbidities, medications, preferences and response to a time-limited monitored trial.

In conclusion, food supplements must not replace core OA treatments. However, patients will continue to ask about them. Rather than avoiding these conversations, healthcare professionals should be equipped to talk about them confidently, consistently and using the best available evidence. We have proposed a practical framework to support these conversations in routine clinical practice.

## Figures and Tables

**Table 1:** Practical summary of the main and most studied nutritional strategies for managing osteoarthritis.

Nutritional Strategy	Main Proposed Mechanism of Action	Authors’ Narrative Interpretation of the Evidence *	Typical Dose Studied **	Expected Time to Onset of Effects ***	Key Safety Considerations	Practical Clinical Message
Healthy, balanced diet and reduction of excess body fat	Categories 1 and 2	Moderate to strong for weight management in overweight/obese OA; indirect for diet	Personalised	For weight loss, it depends on starting weight, but generally takes months	Generally safe when supervised	Nutritional priority. Should be addressed before supplement use, particularly when excess body weight, poor diet quality, or metabolic risk factors are present.
Omega-3 fatty acids from fish, krill, or algae oil	Category 1	Low but promising	1–3 g/day EPA+DHA	Around 3 months	Possible interactions with anticoagulant/antiplatelet therapy	May be discussed as an adjunct, especially in patients with low oily fish intake. Algae-derived sources are suitable for patients avoiding animal-derived products.
Vitamin D	Categories 3 and 4	Low for OA symptoms: stronger rationale when deficiency is present	Personalised based on baseline concentration and form	Around 3 months	Justified when a deficiency is present; monitoring of circulating levels is necessary	Most rational when deficiency, low intake, limited sun exposure, or increased risk of deficiency is present. Not an OA-specific treatment for all patients.
Protein and amino acids	Category 4	Low for OA-specific outcomes; stronger rationale for sarcopenia or frailty	Personalised based on dietary intake, body weight, and clinical condition	Weeks to months	Consider total protein intake, hydration, and clinical contraindications	Most relevant for older, frail, or sarcopenic patients, where supporting muscle function may indirectly support mobility and joint function.
Turmeric extract (curcumin)	Category 1	Low but promising	80–1500 mg/day	Around 3 months	Caution in case of anticoagulant/antiplatelet therapy, biliary diseases, and gastrointestinal intolerance	May be discussed as an adjunct for symptom relief, but formulation and bioavailability vary substantially.
Ginger extract	Category 1	Low	500–1000 mg/day	Around 3 months	Gastrointestinal disorders in some subjects	May be discussed as an adjunct, particularly for patients interested in botanical options, but tolerability should be reviewed.
*Boswellia serrata* extracts	Category 1	Low but promising	100–400 mg/day	Around 3 months	Little documented; consider allergies	Among the more promising botanical options for pain, but evidence remains limited and formulation-dependent.
Collagen preparations (undenatured type II collagen, hydrolysed collagen, collagen peptides)	Category 2	Low but promising	40 mg/day (undenatured type II) or 2–10 g/day (hydrolysed)	3–6 months	Little documented; generally safe	May be discussed as an adjunct. Potential benefit may be more plausible in earlier-stage OA, although this requires further confirmation.
Glucosamine	Category 2	Low; mixed findings	1500 mg/day	3–6 months	Possible drug interactions (anticoagulants) and safety concerns (impaired glucose control)	Evidence is inconsistent. A time-limited monitored trial may be reasonable if no contraindications are present.
Chondroitin sulphate	Category 2	Low; mixed findings	800–1200 mg/day	3–6 months	Little documented; caution with anticoagulant/antiplatelet therapy	Evidence is inconsistent. May be considered as an adjunct, but expectations should be modest, and response should be monitored.
Probiotics, particularly *Lactobacillus* strains	Category 1	Low; emerging	Strain-specific	Weeks to months	Generally safe	Evidence is preliminary and strain-specific. Routine use for OA cannot yet be recommended, but selected use may be discussed.

* “Low” means little evidence, “Low but promising” means limited evidence with generally favourable findings, “Low; mixed findings” means limited evidence with inconsistent findings, and “Low; emerging” means little evidence accompanied by growing interest. ** doses are indicative only and reflect ranges reported in clinical studies or commercial products; formulation, standardisation, and bioavailability may substantially influence the effective dose. *** timing reported is for guidance only.

## Data Availability

No new data were created or analysed in this study. Data sharing is not applicable to this article.

## Appendix A. The 5 A’s of Food Supplement Conversations: A Practical Framework for Discussing with Patients with Osteoarthritis

Patients with osteoarthritis frequently ask whether food supplements such as collagen, glucosamine, or turmeric are “worth taking”. Rather than providing a simple “yes” or “no” answer, clinicians can use the 5 A’s to structure a patient-centred discussion.

⠀

ASK—Understand the patient’s trigger and goal.

Begin by exploring why the patient is asking. Ask who or what has prompted the question (family, friends, social media, advertising, another healthcare professional, dissatisfaction with their clinical condition, etc.). This often reveals the patient’s expectations and provides an opportunity to correct disinformation. Useful questions include:

- Who or what prompted you to ask about this food supplement?
- What are you hoping it will achieve?
- Has someone recommended it to you?
- Are you already taking it?

Understanding whether the patient is seeking pain relief, slowing disease progression, improving mobility, or simply “trying everything possible” helps guide the discussion and manage expectations.

⠀

ASSESS—Reinforce the fundamentals first.

Before discussing food supplements, establish whether the evidence-based foundations of osteoarthritis management are already in place. Consider:

- Regular exercise and strengthening;
- A healthy, balanced dietary pattern (preferably Mediterranean-style);
- Maintaining a healthy body weight;
- Appropriate pain management where required.

A useful phrase is “Supplements should complement, not replace, the treatments we already know are effective.”

⠀

ADVISE—Explain the evidence honestly.

Summarise the current evidence in clear, balanced language. Examples include:

- “Healthy diet and weight management remain the nutritional priority and have the strongest evidence for improving symptoms and overall health.”
- “There is promising evidence for symptom improvement when using omega-3 fatty acids, and supplementation is particularly reasonable in patients with low oily fish intake.”
- “Vitamin D supplementation should be considered when deficiency or increased risk of deficiency is present.”
- “Protein supplementation aimed at improving muscle health may support joint function, and is most relevant for older adults, particularly those with frailty or those with sarcopenia.”
- “Curcumin, *Boswellia* and ginger are all promising botanical options for pain relief, although evidence remains limited.”
- “Growing evidence suggests symptom improvement in some patients using collagen, particularly earlier in the disease.”
- “There is mixed evidence for glucosamine and chondroitin: some patients report worthwhile benefit, others do not.”
- “For probiotics, there is emerging evidence only.”

Emphasise that supplements are adjuncts, not disease cures, and that responses vary between individuals.

⠀

APPRAISE—Consider appropriateness and safety.

Before recommending a food supplement, consider whether it is appropriate for that individual. Review:

- Current medications for food–drug interactions (e.g., anticoagulants);
- Comorbidities (e.g., asthma, gastrointestinal diseases);
- Allergies;
- Product quality and regulation;
- Financial cost;
- Patient preferences.

Explain that food supplements are not free from contraindications, that they may cause side effects or interact with medications, and that product quality can vary between manufacturers. Where appropriate, advise patients to choose reputable, quality-assured products. Also explain that several weeks or months may be required before any benefit becomes apparent.

⠀

AGREE—Decide on a monitored trial.

If the patient wishes to proceed,

- Agree on the food supplement to be used;
- Discuss the expected timescale (typically around 3 months);
- Identify meaningful outcomes to monitor (e.g., pain, function, stiffness);
- Plan follow-ups, arrange clinical review, and discontinuation if there is no clinically important improvement or side effects.

A time-limited monitored trial is more appropriate than indefinite supplementation.

⠀

***Examples of practical responses.***

- Question 1: “Should I take collagen?”
  Answer 1: “Collagen appears safe, and there is promising evidence that it may improve symptoms in some people. If you decide to try it, think of it as an addition to your exercise programme rather than a replacement for it.”
- Question 2: “I’ve heard turmeric is good for arthritis. Should I take it?”
  Answer 2: “Curcumin may reduce pain in some patients, although the evidence is still evolving. If you decide to try it, let’s review whether it is helping after about three months.”
- Question 3: “Should I take glucosamine?”
  Answer 3: “The evidence is mixed. Some people find it helpful, while others do not. If you choose to try it, we should also check that it doesn’t interact with your other medications”
- Question 4: “Do I need vitamin D?”
  Answer 4: “Vitamin D is most useful if you are deficient or at increased risk of deficiency. It isn’t routinely needed for everyone with osteoarthritis.”

⠀

***Key clinical message:*** Every discussion about food supplements is an opportunity to reinforce patient self-management. Food supplements may provide additional benefit for selected patients, but they must always be considered alongside regular exercise, healthy eating and weight management, not as replacements for these core interventions.
